# Methicillin-Resistant *Staphylococcus aureus* in Poultry

**DOI:** 10.3201/eid1503.080696

**Published:** 2009-03

**Authors:** Davy Persoons, Sebastiaan Van Hoorebeke, Katleen Hermans, Patrick Butaye, Aart de Kruif, Freddy Haesebrouck, Jeroen Dewulf

**Affiliations:** Ghent University, Merelbeke, Belgium (D. Persoons, S. Van Hoorebeke, K. Hermans, P. Butaye, A. de Kruif, F. Haesebrouck, J. Dewulf); Veterinary and Agrochemical Research Centre, Brussels, Belgium (P. Butaye); 1 These authors contributed equally to this article.

**Keywords:** Methicillin-resistant Staphylococcus aureus, MRSA, poultry, broiler, laying hen, spa type, MLST, dispatch

## Abstract

Methicillin-resistant *Staphylococcus aureus* (MRSA) has been detected in several species and animal-derived products. To determine whether MRSA is present in poultry, we sampled 50 laying hens and 75 broiler chickens. MRSA was found in some broiler chickens but no laying hens. In all samples, *spa* type t1456 was found.

*Staphylococcus aureus* is a well-known pathogen of humans and animals. Methicillin resistance in this bacterial species represents a threat to human health. Originally, methicillin-resistant *S. aureus* (MRSA) was a nosocomial pathogen, but in the 1990s, MRSA spread into communities worldwide.

Recently, pigs were shown to be a major reservoir for MRSA multilocus sequence type 398 (ST398). Because this sequence type has also been isolated from other animal species, it is referred to as livestock-associated MRSA (*1*). It has also shown potential for zoonotic transmission (*2*). Among ST398 isolates, a variation in *spa* types has been found (*3*). MRSA has been isolated from raw chicken meat or carcasses in Korea (*4*,*5*) and Japan (*6*); however, these strains were human-associated and not the livestock-associated strains. Thus, the possibility of human contamination of poultry carcasses by slaughterhouse employees cannot be ruled out. We investigated whether livestock-associated MRSA is present in commercial broiler chickens and laying hens.

## The Study

In 2007, from randomly selected farms in Belgium, we sampled 5 laying hens from each of 10 farms and 5 broiler chickens from each of 14 farms. One broiler farm was sampled twice (4 months apart, from different flocks in the same house, leaving 1 production round unsampled). Samples were taken from the cloaca and nasal cavity of these 50 laying hens and 75 broiler chickens.

Samples were first incubated in a brain–heart infusion broth supplemented with nalidixic acid and colistin, each at a concentration of 10 µg/mL. After overnight incubation at 37°C, 1 µL of this broth was streaked onto an MRSA ChromID plate (bioMérieux, Marcy l’Etoile, France) and incubated for 24–48 h at 37°C. To differentiate phenotypes of *S. aureus*, we purified colonies that showed typical growth on MRSA Chrom-ID plates by transferring them to modified Baird-Parker agar (*7*) and Columbia blood agar (Oxoid, Hampshire, UK) for DNAse and catalase testing.

 The phenotypically identified MRSA strains were then confirmed by 16S rRNA-*mecA*-*nuc* triplex PCR as previously described (*8*). For all strains, the spa type was determined, and for 3 strains, multilocus sequence typing (MLST) was performed as described (*9*). Relatedness with other *spa* types from porcine origin (*10*) was determined by using Ridom SpaServer software version 1.3 (Ridom GmbH; Würzburg, Germany; www.ridom.de/spa-server).

Disk susceptibility of the strains was tested by using the Kirby-Bauer disk-diffusion method. Clinical Laboratory Standards Institute guidelines (M31-A3) were followed for inoculum standardization. After plates were incubated for 18 h, inhibition zones were measured in millimeters and interpreted according to Neo-Sensitabs manufacturer’s instructions (http://rosco.dk). *S. aureus* ATCC 25923 was included for internal quality control.

MRSA was not isolated from any laying hen samples. This finding may indicate that MRSA is absent or present only in low numbers in laying hens, possibly because of the limited use of antimicrobial drugs in these animals. Use of certain antimicrobial drugs in human hospitals has been shown to be a risk factor for acquiring MRSA infection, especially when the chosen treatment is inappropriate or insufficient (*11*). Antimicrobial-drug use may also be a risk factor for MRSA colonization of animals. The antimicrobial drugs used in the flocks included in this study were tylosin, amoxicillin, trimethoprim-sulfamethoxazole, lincomycin, tetracycline, and colistin.

MRSA was isolated from 8 broiler chickens from 2 of the 14 farms sampled. Low prevalence in poultry has also been found by Kitai et al. (*6*) and Lee (*5*), although they sampled chicken carcasses from slaughterhouses and did not find any livestock-associated strains. Given our relatively small sample size, our data did not permit us to estimate the within- and between-flock prevalence.

In the MRSA-positive flocks, the number of positive samples varied between 1/5 (20%) and 5/5 (100%). From the 1 MRSA-positive farm that was sampled twice, MRSA was isolated on both occasions. This finding indicates that MRSA may persist on a farm and colonize future flocks. MRSA was found in nearly equal numbers from the nares samples and the cloaca samples. Of the 8 MRSA-positive animals (16 samples), MRSA was found in all samples except for 1 cloacal swab, for a total of 15 MRSA isolations.

Susceptibility testing showed that all 15 isolated strains were resistant to erythromycin, kanamycin, tobramycin, lincomycin, tylosin, tetracycline, and trimethoprim. All strains were susceptible to chloramphenicol, ciprofloxacin, linezolid, mupirocin, quinopristin-dalfopristin, rifampin, and sulfonamides.

Molecular typing showed that the strains all belonged to *spa* type t1456 of the livestock-associated ST398, which is typically not typeable by pulsed-field gel electrophoresis. To our knowledge, this *spa* type has not been found in other animal species (*11*,*12*). Its relatedness to other *spa* types isolated from pigs is shown in the Table. A shortening of variable number tandem repeat composition seems to be present in *spa* type t1456. Whether t1456 is a clone typically associated with poultry, or specifically broiler chickens, and whether it is spreading internationally needs further investigation.

**Table:** Comparison of *spa* type methicillin-resistant *Staphylococcus aureus* isolated from pigs and poultry*

Source	*spa* type	Composition†
Pig	t011	008 – 16 – 02 – 25 – – – – – 34 – 24 – 25
Pig	t034	008 – 16 – 02 – 25 – 02 – 25 – 34 – 24 – 25
Pig	t108	008 – 16 – 02 – 25 – – – – – – – 24 – 25
Pig	t567	008 – – – 02 – 25 – – – – – – – 24 – 25
Pig	t943	008 – 16 – 02 – 25 – – – 25 – – – 24 – 25
Pig	t1254	106 – 16 – 02 – 25 – – – – – 34 – 24 – 25
Pig	t1255	008 – 16 – – – – – – – – – 34 – 24 – 25
Poultry	t1456	008 – 16 – 02 – 25

*All *spa* types isolated from pigs were described in de Neeling AJ, van den Broek MJM, Spalburg EC, van Santen-Verheuvel MG, Dam-Deisz WDC, Boshuizen HC, et al. High prevalence of methicillin-resistant *Staphylococcus* *aureus* in pigs. Vet Microbiol. 2007;122:366–72 †Variable number tandem repeat composition in the 3’ end of the *spa* gene.

## Conclusions

We confirmed the presence of MRSA in broiler chickens, but we were unable to find it in laying hens. All isolates belonged to 1 *spa* type, t1456, and thus differed from the other strains belonging to ST398 isolated from other animal species in Belgium and abroad. Whether this *spa* type is typically associated with poultry still needs to be confirmed. More detailed data are also needed to gain further insight in the true within- and between-flock prevalence of MRSA in poultry and its evolution over time.
