# Erratum—Vol. 15, No. 3

**DOI:** 10.3201/eid1505.999000

**Published:** 2009-05

A reference was missing from the article Methicillin-Resistant *Staphylococcus aureus* in Poultry (D. Persoons et al.). In the Table accompanying the article, the data on *spa* types isolated from pigs were originally described in de Neeling AJ, van den Broek MJM, Spalburg EC, van Santen-Verheuvel MG, Dam-Deisz WDC, Boshuizen HC, et al. High prevalence of methicillin-resistant *Staphylococcus aureus* in pigs. Vet Microbiol. 2007;122:366–72. The article has been corrected online (www.cdc.gov/EID/content/15/3/452.htm).

